# The physiochemical properties and shelf‐life of orange‐fleshed sweet potato puree composite bread

**DOI:** 10.1002/fsn3.710

**Published:** 2018-07-11

**Authors:** Cecilia Wanjuu, George Abong, Daniel Mbogo, Simon Heck, Jan Low, Tawanda Muzhingi

**Affiliations:** ^1^ Department of Food Science, Nutrition and Technology University of Nairobi Kangemi Kenya; ^2^ Food and Nutritional Evaluation Laboratory (FANEL) CIP‐SSA Biosciences east and Central Africa (BecA) ILRI Nairobi Kenya; ^3^ International Potato Centre (CIP), Sub‐Saharan Africa (SSA) Regional Office Nairobi Kenya

**Keywords:** β‐carotene, color, microbial, puree bread, texture, water activity

## Abstract

Value addition of orange‐fleshed sweet potato (OFSP) increases diversified utilization of this crop, which is rich in provitamin A carotenoids. OFSP bread, in which 30%–45% of wheat flour is replaced with OFSP puree, has been successfully commercialized in Kenya. However, the effect of this substitution on the bread's physiochemical properties and its shelf‐life are currently unknown. This study was designed to determine the physiochemical properties and shelf‐life of OFSP puree‐wheat flour composite bread (30% puree), compared to standard, 100% wheat flour, bread. Freshly baked bread samples were stored at 7, 20, 25, and 30°C, and monitored for moisture content, water activity, color, texture, volume, carotenoids, and microbial load. The moisture content, β‐carotene content, and color of bread significantly decreased with increase in storage temperature and time (*p* < 0.05). Bread made with OFSP puree had a longer shelf‐life, showing spoilage on day six compared with the white bread, which spoiled on the fourth day. This is attributed to the significantly higher water activity in white bread than in the OFSP bread. The substitution of wheat with OFSP puree resulted in reduced extensibility of gluten, thus, specific volume of white bread was significantly (*p* < 0.05) higher than that in OFSP puree bread. Refrigeration increased crumb firmness, chewiness and cohesiveness in both types of bread. In conclusion, OFSP puree increases the water binding capacity of the bread, which reduces water activity and increases its shelf‐life.

## 1 INTRODUCTION

The orange‐fleshed sweet potato (OFSP) is an essential source of provitamin A carotenoids, energy, and nutrients, such as minerals and dietary fiber (Kapinga et al., 2005; Low & Jaarsveld, 2008). OFSP is now recognized as an effective and sustainable food‐based dietary intervention strategy to address vitamin A deficiency (VAD) in developing countries through its incorporation into the diets at household level (Kapinga et al., 2005). OFSP is commonly consumed fresh, boiled, or roasted. More recently, many households are processing OFSP into flour or mashed into puree to substitute wheat flour in chapatti and mandazi (doughnuts) (Muoki & Agili, 2015; Stathers et al., 2013). Many farms also get income from it by selling it to high‐end fresh markets in urban centers. While sweet potato and OFSP processing in China is highly advanced and commercialized, sweet potato processing in sub‐Saharan African (SSA) countries, such as Kenya is slowly being introduced. This is partly because sweet potato is traditionally considered a poor man's food and is grown by women in marginal lands (Andrade et al., 2009).

The International Potato Center (CIP) has gained considerable experience by pilot work in Rwanda and has partnered with companies manufacturing and marketing bakery products in which 20%–45% of wheat flour has been replaced by OFSP puree (boiled and mashed roots). Experience across many SSA countries has shown that with the current price of sweet potato roots, it is economically advantageous to use OFSP puree as a wheat substitute instead of OFSP flour (Awuni, Alhassan and Amagloh, 2017). Moreover, products made using OFSP puree are highly acceptable to consumers. Some large supermarkets in Kenya are currently using OFSP puree as a wheat flour substitute in their bakeries to make bread, buns, muffins, and cookies. The bakeries use OFSP puree to bake bread, buns, and scones with up to 30%–50% wheat substitution (Muoki & Agili, 2015; Stathers et al., 2013). The bakeries benefit from using OFSP puree in two ways: First, their production costs are lowered using a locally grown ingredient and secondly, the OFSP puree yields an innovative, healthier bread with increased consumer demand.

The quality and shelf‐life of bread depend on the ingredients used in baking (Onyango, 2016). Bread starts to deteriorate immediately after baking due to chemical and physical changes as described in the staling process (Gray & Bemiller, 2003). The redistribution and migration of water, gluten transformation, and starch retrogradation are the main causes of staling in bread (Choi, 2010). Staling affects the texture of the bread by increasing the hardness, crumbliness, and opacity. The aroma and taste of bread also changes (Purhagen, Sjöö and Eliasson, 2012). Substitution or replacement of wheat flour influences the textural, nutritional, and organoleptic properties and hence the shelf‐stability of the bread. A lot of research has been performed on the physiochemical characteristics of bread (Adebayo‐Oyetoro, Ogundipe, & Adeeko, 2016). (Aleid, Al‐Hulaibi, Ghoush, & Al‐Shathri, 2015; Arendt, Ryan, & Dal Bello, 2007; Charoenthaikij et al., 2010; Gellynck, Kühne, Van Bockstaele, Van de Walle, & Dewettinck, 2009; Trejo‐González, Loyo‐González, & Munguía‐Mazariegos, 2014). OFSP puree composite bread is a new concept in SSA. Currently, very little is known about its shelf‐keeping qualities, hence its shelf‐life. Novel baking technologies have been used with attempts to extend the shelf‐life of bread and improve on the flavor and texture to better respond to the dynamic market demands (Corsetti, Martin, & Pesenti, 2007). One of the important factors affecting bread shelf‐life is water activity (Jay, Loessner, & Golden, 2005; Nielsen, 2010). The ability of mold to grow is characterized by the water activity of the food. Low water activity (>0.85) is significant in the preservation of foods (Jay et al., 2005). Baked products have a water activity of 0.75 – 0.95 and hence spoil due to mold growth (Smith, Daifas, El‐Khoury, Koukoutsis, & El‐Khoury, 2004). Several mold genera have been found to causes bread spoilage, those of importance being *Eurotium, Penicillium,* and *Aspergillus* (Saranraj and Sivasakthivelan, 2015). Therefore, it is important to establish the physiochemical and textural properties of OFSP puree bread and to determine its shelf‐life to increase its economic value and impact. Therefore, this study was designed to investigate the effect of storage temperature on moisture content, water activity, microbial growth, color and carotenoid content, texture, and volume in OFSP puree‐based bread compared with standard white bread while considering current storage practices in Kenya. This is crucial to the food industry as consumers not only demand for healthy and nutritious foods but also products with an extended shelf‐life that do not have the potential to cause illness.

## 2 MATERIALS AND METHODS

### 2.1 The process of bread baking

**Figure 1:**
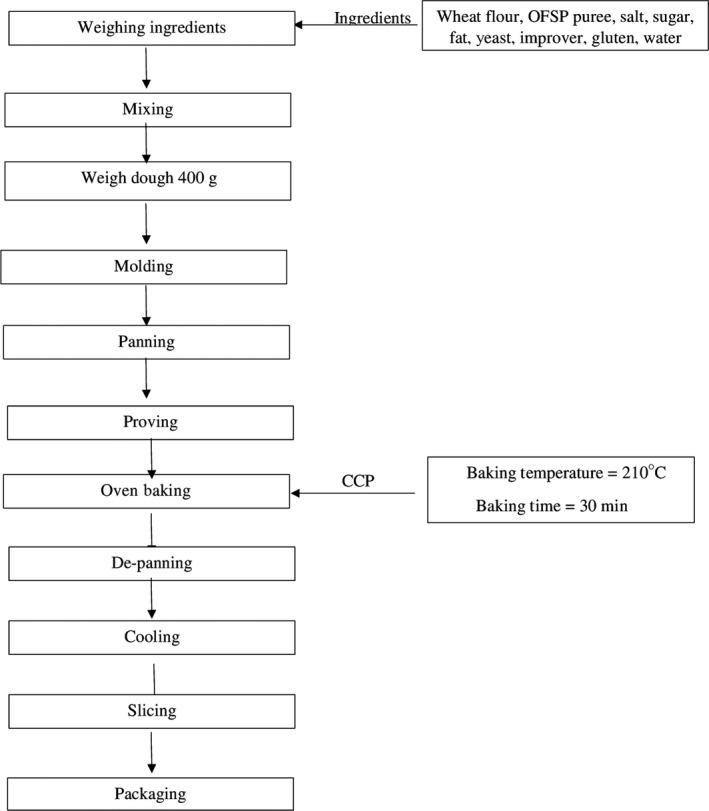
Tuskys bread‐baking process

The process of bread baking in Tuskys bakery is as shown in Figure 1. The ingredients (Table 1) are weighed and mixed for 5 min in a Tombak bakery and confectionary F2 mixer. The baking conditions and duration for the OFSP puree bread and the white bread are the same. The dough is then cut and weighed into 460 g portions and then molded into rolls which are placed in bread pans. The dough is allowed to proof for 45 min in a proofing cabinet. This allows for the dough to relax and yeast to produce carbon IV oxide allowing the dough to rise to a desirable loaf volume (Onyango, 2016). The dough is then baked in a FİMAK Rotary Oven at 210°C for 30 min. During baking, heat transfer occurs by conduction, radiation, and convection (Anishaparvin, Chhanwal, Indrani, Raghavarao, & Anandharamakrishnan, 2010). The loaves of bread are depanned and allowed to cool to room temperature. The loaves are then sliced and packaged for distribution.

**Table 1:** Ingredients of bread baking at Tuskys as a percentage

Ingredients/products	OFSP puree bread	White bread
Flour	48.17	71.80
Sugar	1.78	4.04
Salt	0.80	1.01
Fat	2.32	2.69
Yeast	0.71	0.27
OFSP puree	31.22	–
Improver	0.36	–
Gluten	0.36	–
Water	14.27	20.19

*Note*

^*^No preservatives were added to the breads according to the manufacturer procedures.

### 2.2 Sample collection

The bread samples were collected fresh from the oven in brown bags from Tuskys, and were shipped in cooler boxes to the Food and Nutritional Evaluation Laboratory (FANEL), CIP‐SSA, Biosciences east and central Africa (BecA). The analysis was performed on the first day (baseline), and then the samples were stored at 7°C (in a cold room), and in incubators set at 20, 25, and 30°C. Samples were subsequently analyzed at end line. These conditions were monitored throughout the analysis period using a stainless steel temperature probe. The following analysis was conducted.

### 2.3 Determination of water activity

Water activity was measured using a water activity meter Aqua Lab Series 4TE from Decagon Devices, Inc. (NE Hopkins Ct. Pullman, WA, USA). The water activity meter was calibrated using the verification standards (aw = 0.765 and aw = 0.500) at 25°C, and the values were determined to be within the required range of ±0.001. The water activity of the bread samples was measured in triplicates (Juan‐Borras, Soto, Gil‐Sanchez, Pascual‐Mate, & Escriche, 2016).

### 2.4 Determination of moisture content

Moisture content was determined in triplicates using the Mettler Toledo HE53 Halogen Moisture Analyzer (Switzerland). The sample was weighed (2 g), heated using halogen to evaporate all moisture, and then the moisture content of the original sample was calculated based on the weight loss and displayed real time on the moisture balance digital display.

### 2.5 Microbial analysis

Total viable bacterial counts (TVC) and fungal (yeast and molds) were analyzed in both the OFSP and white bread samples as described in AOAC 2012. Briefly, bread samples were prepared by homogenizing 10 g in buffered peptone water, and then, 1 ml of this was used to make subsamples decimally diluted up to four dilutions. Pour plate method was used during plating (Jay et al., 2005). This was done in triplicates. The TVC plates were incubated at 30°C for 4 days, while those of yeast and mold were incubated at 25°C for 5 days.

### 2.6 Carotenoid analysis

Carotenoids were determined as described by Kurilich and Juvik (1999) with some minor modifications. All extraction of carotenoids from the breads was performed under golden lights in Food and Nutritional Evaluation Laboratory (FANEL), CIP‐SSA, Biosciences east and central Africa (BecA). Briefly, 1 g of bread (crumb or crust) underwent a 5‐min ethanol precipitation (6 ml of ethanol containing 0.1% butylated hydroxytoluene (BHT) in an 85°C‐water bath before being subjected to a 10‐min saponification with 120 μl of 80% w/v KOH in water. Bread contains significant amounts of fat from shortening; therefore, saponification is necessary to hydrolyze the fatty acid esters of carotenoids, which eases extraction (Kopec, Cooperstone, Cichon and Schwartz, 2012). All samples were vortexed once during saponification. Upon removal, they were immediately placed in an ice bath where 3 ml of distilled deionized water and 3 ml of hexane were added. The mixture was vortexed for 30 s and centrifuged at 800× g for 5 min. The hexane fraction was transferred into a 15‐ml test tube and the extraction repeated four times with 3 ml of hexane. The hexane fractions were dried completely under a gentle stream of nitrogen gas using a N‐Evap System (Organomation, Berlin, MA). The dried residue was reconstituted in 3 ml of hexane and washed with 3 ml distilled deionized water, vortexed for 30 s, and centrifuged at 800× g for 5 min. The hexane top layer was collected into a 5‐ml test tube and dried under a gentle stream of nitrogen using an N‐Evap system. The dried test tube contents were reconstituted in 1 ml of ethanol, sonicated for 1 min, vortexed for 30 s, and transferred into a 2 ml HPLC vial. Then, 50 μl was injected into the HPLC system for analysis.

The HPLC systems consisted of a Shimadzu CBM ‐20A Prominence Bus Module, SPD –M20A Prominence Photo Diode Array (PDA), DGU 20A5R Prominence Degasser Module, SIL 30AC Nexera Autosampler, two Nexera X2 LC 30AD pumps, a YMC Carotenoid S‐3 μm, 150 × 3.0 mm I.D column, and Shimadzu LabSolutions data management software. The HPLC solvent gradient elution and time program were used as previously published (Yeum et al., 1996). The HPLC mobile phase was methanol: methyl‐tert‐butyl ether: water (83:15:2, v/v/v, with 1.5% ammonium acetate in the water, solvent A) and methanol: methyl‐tert‐butyl ether: water (8:90:2, v/v/v, with 1% ammonium acetate in the water, solvent B). The gradient procedure at a flow rate of 1 ml/min was as follows: (a) 90% solvent A and 10% solvent B for 5 min; (b) a 12‐min linear gradient to 55% solvent A; (c) a 12‐min linear gradient to 95% solvent B; (d) a 5‐min hold at 95% solvent B; and (e) a 2‐min gradient back to 90% solvent A and 10% solvent B. Carotenoids were monitored at UV maximum absorption of 450 nm, and DAD spectral data from 250 to 550 nm were stored to examine spectrum peaks for carotenoids. Carotenoids were quantified by determining peak areas in the HPLC chromatograms calibrated against known amounts of standards. Concentrations were corrected for extraction and extraction and handling losses by monitoring the recovery of the internal standard (Echinenone).

### 2.7 Bread crust and crumb color

Color determination was carried out on breadcrumb and crust using Lovibond^®^ LC 100/SV 100 handheld spectrocolorimeter (England), and results were expressed in accordance with the Hunter Lab color CIE L*a*b space. The parameters determined were L* (light/dark) a* (=red/green), b* (blue/yellow), and C* (bright/dullness) (Mannuramath, Yenagi, & Orsat, 2015). The standard used in this study was the bread color during baseline analysis.

### 2.8 Bread volume

Bread volume was determined by displacement method using bird millet as previously described (AACC, 2001). Specific volume was calculated as volume to weight ratio (cm^3^/g) (Mannuramath et al., 2015).

### 2.9 Crumb firmness

Texture profile analysis was performed using a texture analyzer (TA‐XT2, Stable Micro Systems, Godalming, United Kingdom) equipped with a 36‐mm Perspex cylinder probe along with a 50‐kg load cell according to AACC Method 74‐10A (2000). The Texture Analyzer measured the primary parameters; hardness, springiness, cohesiveness, and resilience, and secondary characteristics; gumminess; and chewiness.

### 2.10 Statistical analysis

Data analysis was performed using GenStat 15th Edition. Data collected were subjected to analysis of variance (ANOVA) and the means separated by Duncan multiple range test. The significance level was set at *p* = 0.05.

## 3 RESULTS AND DISCUSSION

### 3.1 Moisture content of OFSP and white bread

Orange‐fleshed sweet potato puree bread had a higher moisture content, ranging between 30.20% and 33.38%, compared to a range of 29.02% to 30.15% for standard white bread (Table 2). There was a significant decrease in moisture content of bread with increase in storage temperature for both OFSP puree bread and standard white bread (*p* < 0.05). Previous studies (Gray & Bemiller, 2003) showed that bread stored at lower temperatures dried out quickly, accelerating the rate of staling. Staling is correlated with recrystallization of starch. Starch crystallization diminished with increase in storage temperature, which also resulted in staling of bread. These findings agree with previous studies (Abu‐Ghoush et al., 2007) that concluded that moisture content significantly decreased after storage, ultimately resulting in firmer composite bread.

**Table 2:** Moisture content and water activity in OFSP and white bread

Parameter	Storage temperature	Moisture content				Water activity			
Sample		7°C	20°C	25°C	30°C	7°C	20°C	25°C	30°C
OFSP puree‐wheat flour composite bread	Baseline	33.88^i^	31.89^h^	30.55^g^	30.20^fg^	0.9361^cd^	0.9296^fg^	0.9260^bc^	0.9254^bc^
	End line	28.93^cd^	29.81^ef^	27.44^ab^	29.45^de^	0.9377^de^	0.9338^gh^	0.9362^f^	0.9416^hi^
White bread	Baseline	29.02^cd^	30.15^fg^	29.71^ef^	29.69^ef^	0.9248^ef^	0.9206^ab^	0.9273^bcd^	0.9352^a^
	End line	27.15^a^	27.22^a^	27.81^b^	28.51^c^	0.9290^fgh^	0.9248^bcd^	0.9457^i^	0.9541^fgh^

*Note*

All values reflect mean counts. Values bearing different superscript letters in each row are significantly different (*p*<0.05)

### 3.2 Water activity in OFSP and white bread

There were significant differences (*p* < 0.05) in the water activities of OFSP puree bread (0.9207) and white bread (0.9320) at baseline. Increased storage temperature affected resulted in higher water activity (Table 2). The white bread had significantly (*p* < 0.05) higher increase in the water activity than the OFSP bread. It is notable that the white bread had significantly higher (*p* < 0.05) change in water activity level compared with the OFSP bread, favorable for mold growth (Abu‐Ghoush et al., 2007). The decrease in water activity in the OFSP puree bread was possibly due to concentration of sugars attributed to the use of OFSP puree (Jay et al., 2005). According to Nielsen (2010), the less available the water activity, the fewer chemical reactions, and microbial growth cause decomposition.

### 3.3 Microbial load in OFSP puree and white bread

Initially, no colonies were observed for total microbial and fungal counts for both the OFSP and white bread. This indicates that the loaves of bread were not contaminated, as baking temperatures are effective in destroying contaminants from the ingredients and general handling. However, contamination could arise following baking, depending on how the bread is handled after baking, during cooling, and throughout packaging, distribution, and storage. No microbial counts were observed for either bread after storage at 7°C and 20°C. However, by the end line analysis, total microbial counts in OFSP puree bread increased to 2.88 × 10^3^ and 3.24 × 10^3^ CFU/g after storage at 25 and 30°C, respectively (Table 3). White bread stored at refrigeration had no microbial growth, suggesting that refrigeration preserved the white bread. Total microbial counts in white bread increased from 3.68 × 10^3^ at 20°C, to 3.93 × 10^3^ and 4.25 × 10^3^ CFU/g, at 25 and 30°C, respectively. The significant difference (*p* < 0.05) in total counts may have been due to differences in storage temperature, moisture, and water activity levels (Ayub, Dahab, & Durran, 2003).

**Table 3:** Microbial load in OFSP and white bread

Sample	Storage temperature	TVC		Yeast and mold	
Microbial analysis		Baseline	End line	Baseline	End line
OFSP puree bread	7°C	–	–	–	–
	20°C	–	–	–	–
	25°C	–	2.88 × 10^3^ CFU/g	–	–
	30°C	–	3.24 × 10^3^ CFU/g	–	–
White bread	7°C	–	–	–	–
	20°C	–	3.68 × 10^3 ^CFU/g	–	2.19 × 10^3^ CFU/g
	25°C	–	3.93 × 10^3^ CFU/g	–	3.94 × 10^3^ CFU/g
	30°C	–	4.25 × 10^3^ CFU/g	–	3.97 × 10^3^ CFU/g

*Note*

–: Represents no growth.

There was no fungal growth on the OFSP puree bread at different storage conditions during the 7‐day study period (Table 3). White bread stored in refrigeration temperatures did not have fungal growth during the study period. However, fungal growth was observed on the white bread stored at 20, 25, and 30°C with colony counts of 2.19 × 10^3^, 3.94 × 10^3^, and 3.97 × 10^3^ CFU/g, respectively. This is beyond the acceptable limit for Kenya Standards for bread that allows for 100 CFU/g (KS 172:2010). The high microbial population in the white bread could be due to its moisture content and water activity levels, which were significantly different (*p* < 0.05) from microbial counts in OFSP bread. These parameters are known to provide favorable conditions for growth (Nielsen, 2010). Microbial growth was visible on the fourth and sixth days for white and OFSP bread, respectively.

### 3.4 Carotenoid content

**Figure 2:**
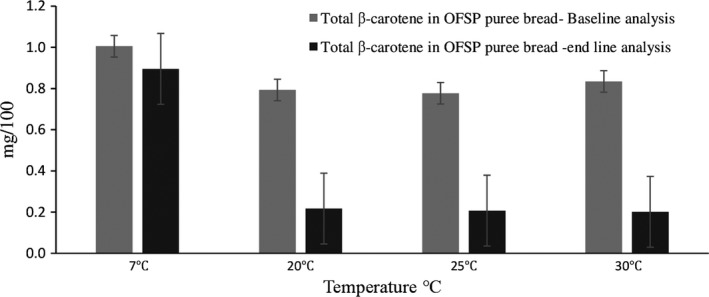
β‐carotene content of orange‐fleshed sweet potato (OFSP) bread

Refrigerating OFSP puree bread preserved β‐carotene content with minimal loss of carotenoids. Storage of bread at temperatures above 20°C resulted in loss of carotenoids (Figure 2), because carotenoids are prone to oxygen and light‐thermal‐dependent deterioration (Yusuf, Fuchs, & Nicolaides, 2015); (De Moura, Miloff, & Boy, 2015). Refrigeration preserved the carotenoids compared with storage at higher temperature. There was no significant difference in the β‐carotene contents during end line analysis for storage at temperatures at 20, 25, and 30°C, respectively. Carotenoids are known to deteriorate when subjected to heat treatment (thermal degradation) (De Moura et al., 2015). Carotenoids were also detected in the white bread and were identified as lutein, zeaxanthin, and β‐cryptoxanthin. These xanthophylls could be coming from the wheat flour (Bhatnagar‐Panwar, Bhatnagar‐Mathur, Bhaaskarla, Dumbala, & Sharma, 2013). The OFSP puree bread had significant (*p* < 0.05) levels of total carotenoids, mostly β‐carotene, that ranged from 0.049 to 0.053 mg/100 g (Figure 2). No β‐carotene was detected in the white bread.

### 3.5 OFSP and white bread crust and crumb color

Increase in storage temperature resulted in a significant (*p* < 0.05) decrease in the L* (lightness/darkness) of both OFSP and white breadcrumb and crust. Storage in refrigeration temperatures did not cause significant changes on the L* values of OFSP and white breadcrumb and crust (Table 4).

**Table 4:** Color analysis of OFSP and white breadcrumb

Sample	Storage temperature	OFSP puree bread				White bread			
		7°C	20°C	25°C	30°C	7°C	20°C	25°C	30°C
*L*	Baseline	73.67^d^	73.80^d^	73.83^d^	73.43^d^	78.47^f^	77.07^e^	77.00^e^	77.33^e^
	End line	70.53^c^	71.47^c^	72.70^d^	66.80^b^	78.70^f^	71.37^c^	71.60^c^	20.23^a^
*a*	Baseline	5.93^fg^	5.83^fg^	6.33^fg^	6.53^g^	−1.67^bc^	−0.57^d^	−1.27^cd^	−1.40^cd^
	End line	4.37^e^	5.57^f^	6.60^g^	6.60^g^	−1.20^cd^	−2.47^b^	−3.47^a^	4.57^e^
*b*	Baseline	31.70^fg^	32.27^g^	31.53^fg^	31.17^fg^	14.50^d^	12.57^b^	12.57^b^	12.57^b^
	End line	27.33^e^	32.13^g^	30.60^f^	30.63^f^	13.17^bc^	14.57^d^	14.57^cd^	0.57^a^
*C*	Baseline	31.90^g^	32.23^g^	31.97^g^	32.07^g^	14.60^e^	12.33^b^	13.63^cde^	12.70^bc^
	End line	27.40^f^	32.70^g^	31.40^g^	31.63^g^	13.33^bcd^	13.23^bcd^	14.43^de^	5.43^a^

*Note*

All values reflect mean counts. Values bearing different superscript letters in each row are significantly different (*p* < 0.05).

The white breadcrumb became darker after 7 days of storage at 30°C with L* values significantly (*p* < 0.05) decreasing from 77.33 to 20.23. The difference in luminosity was because of mold growth on the white bread. Increase in storage temperature resulted in a significant (*p* < 0.05) decrease in the luminosity of the white bread crust and crumb. There was no significant difference (*p* > 0.05) in the luminosity of the OFSP bread crust and crumb due to differences in storage temperature after 7 days. This is due to absence of mold growth on the OFSP bread. Luminosity in the white bread crust significantly (*p* < 0.05) decreased after 7 days' storage at 25 and 30°C (from 63.63 to 57.23). Storage of white bread at 20°C had no significant (*p* > 0.05) effect on the L*values of the crust. The crust of the white and OFSP bread was brown in color due to Maillard reactions: the reaction of reducing sugars with free amino acids in the proteins (Aguilar, Albanell, Minarro, Guamis, & Capellas, 2015). Storage temperature had a significant effect on the redness, (a* values) of the OFSP breadcrumb and crust, a decrease was noted to indicate green color. Storage of the white bread at 30°C resulted in a significant increase (*p* < 0.05) in a* values, and the breadcrumb and crust indicated a red color due to mold growth. A significant (*p* < 0.05) decrease in the b* values was observed in both the OFSP and white breadcrumb and crust after the storage period in the various temperature as recorded in Table 4. White bread stored at 30°C was significantly (*p* < 0.05) affected, becoming a blue color. This can be explained by the presence of mold on the crumb and crust. C* defines the brightness or dullness of a luminous object. There was no significant difference (*p* > 0.05) in the brightness of OFSP with respect to storage temperature at 20, 25, and 30°C. Storage at refrigeration temperatures (7°C) caused a significant decrease on the C* value, and the OFSP breadcrumb was duller. The crumb of the white bread was initially brighter. There was no significant difference (*p* > 0.05) on the C* values between white bread stored at 7 and 20°C. Storage at 30°C had a significant (*p* < 0.5) effect, decreasing the C* value to (5.43); here, the white breadcrumb was dull due to mold growth. The change in redness (a*) and yellowness (b*) of the bread can be linked to the carotenoid content. The degradation of carotenoids caused a change in color (Limbo, Torri and Piergiovanni, 2007).

### 3.6 OFSP and white bread volume

**Figure 3:**
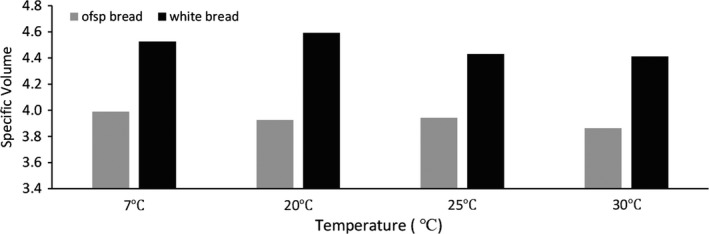
Specific volume of orange‐fleshed sweet potato (OFSP) and white bread

The specific volume of white bread was found to be significantly higher (*p* < 0.05) than that of the OFSP puree bread as shown in Figure 3. The specific volume increased with increase in storage temperature. The specific volume of OFSP bread was significantly lower (*p* < 0.05) ranging between 3.863 and 3.989 cm^3^/g, while that of the white bread ranged between 4.412 and 4.592 cm^3^/g. The lower specific volume in OFSP puree bread may be owed to the reduced extensibility of wheat gluten, due to its partial substitution with OFSP puree (Tsai et al., 2012); (Gewehr, Danelli, Melo, Flöres, & Jong, 2016). The reduction in volume may be due to dilution of gluten, physical interactions, and chemical reactions between fiber components, water, and gluten. Bread volume results from both yeast gassing power and the ability of the gluten matrix to stretch and retain gas. Due to the fiber effect, the air “escapes,” leaving the bread denser with a smaller volume (Gewehr et al., 2016). This aspect influences the texture of the product over its shelf‐life. This can be countered by the addition of gluten or gelatinized starch to the OFSP puree bread to improve the expansion of the pore walls of baked bread for sustainability of gas pressure produced during fermentation and

baking (Tsai et al., 2012). Feili, Zzaman, Abdullah & Yang, (2013) suggested that increasing water content of dough can solve the negative effect of substitution and protein dilution on the loaf volume.

### 3.7 OFSP and white bread texture

Texture analysis results showed an increase in hardness, gumminess, and chewiness and a decrease in springiness, cohesiveness, and resilience after storage in the various temperatures for both the white and OFSP bread (Table 5). It has been reported that refrigeration of bread increases the rate at which the process of crystallization of amylose and amylopectin matrix occurs, which then contributes to overall bread texture and consequently staling. Bread hardness was due to interactions between gluten and fibrous materials, as well as from moisture loss. (Feili, Zzaman, Abdullah, & Yang, 2013; Gray & Bemiller, 2003). OFSP puree bread recovered from its deformed condition (springiness) after the deforming force was removed, at a significantly high rate (*p* < 0.05) when compared to the white bread. This could be attributed to amounts of gluten added in the mixing of OFSP dough, which led to an increase in elasticity of the dough. High values of cohesiveness are desirable in bread, as this leads to less forces required for disintegration during mastication (Onyango, Mutungi, Unbehend, & Lindhauer, 2010). There was no significant difference (*p* > 0.05) in cohesiveness in the OFSP and white bread at the beginning of the experiment. Refrigeration of the bread resulted in a significant increase in cohesiveness as compared to bread stored at 30°C. The OFSP bread was significantly (*p* < 0.05) affected by storage conditions at 30°C compared with the white bread. Increase in gumminess was significantly (*p* < 0.05) higher after storage in refrigeration temperatures than after storage at 30°C. This could be attributed to the increase in hardness caused by crystallization of amylopectin and amylases for both breads. Gumminess in OFSP bread is significantly (*p* < 0.05) higher than in white bread due to dilution of the soluble proteins of the protein matrix embedded in the starch granules. This results in less interference with the starch. Chewiness is a derived

**Table 5:** Texture profile analysis of OFSP and white bread

Samples	Storage temperature	OFSP bread				White bread			
		7°C	20°C	25°C	30°C	7°C	20°C	25°C	30°C
Hardness (*N*)	Baseline	1.472^ab^	1.150^a^	2.169^bc^	2.771^cd^	2.152^bc^	3.424^d^	2.305^bc^	2.456^c^
	End line	4.647^e^	2.501^c^	2.922^cd^	5.217^ef^	6.023^f^	5.223^ef^	5.002^e^	5.500^ef^
Springiness	Baseline	0.9101^abc^	0.8861^ab^	0.9030^abc^	0.9150^abc^	0.9158^abc^	0.9068^abc^	0.9150^abc^	0.9855^de^
	End line	0.8919^ab^	0.8655^a^	0.9525^cd^	0.9051^abc^	0.9051^abc^	0.9216^bc^	0.8911^ab^	1.0144^e^
Cohesiveness	Baseline	0.7817^d^	0.7894^d^	0.8768^e^	0.7680^cd^	0.7724^cd^	0.7592^cd^	0.8759^e^	0.7615^cd^
	End line	0.6286^a^	0.7550^bcd^	0.9480^ef^	0.7172^bcd^	0.7143^bcd^	0.6907^abc^	0.9545^f^	0.6566^ab^
Gumminess	Baseline	1.490^ab^	0.906^a^	1.668^b^	2.368^cde^	1.641^b^	2.431d^e^	1.750^bc^	1.871^bcd^
	End line	2.915^ef^	1.884^bcd^	2.067^bcd^	2.889^ef^	3.956^gh^	3.743^g^	3.446^fg^	4.443^h^
Chewiness	Baseline	1.058^ab^	0.829^a^	1.220^abcd^	1.152^abc^	1.604^bcde^	2.240^fg^	1.502^bcd^	1.657^cde^
	End line	2.598^gh^	1.706^cdef^	2.130^efg^	1.774^def^	3.059^hi^	3.798^j^	3.582^ij^	3.270^ij^
Resilience	Baseline	0.3349^def^	0.3559^f^	0.3296^cdef^	0.4321^g^	0.3163^bcdef^	0.3243^bcdef^	0.3494^ef^	0.4958^h^
	End line	0.2739^ab^	0.3006^bcde^	0.2464^a^	0.6796^i^	0.2796^abc^	0.2719^ab^	0.2831^abcd^	0.9193^j^

*Note*

All values reflect mean counts. Values bearing different superscript letters in each row are significantly different (*p* < 0.05).

attribute of texture and it is a product of hardness, springiness, and cohesiveness and usually represents the energy required to chew solid foods to a desirable state ready for swallowing. OFSP bread had a significant increase (*p* < 0.05) in chewiness at refrigeration temperature due to increase in hardness, springiness, and cohesiveness. Resilience decreased with increase in storage temperature. At temperatures of 30°C, resilience was significantly lost in white bread compared with OFSP puree bread as shown in Table 5.

## 4 CONCLUSION

The use of OFSP puree in bread baking improved the quality of bread as the puree bread exhibited desirable levels for the moisture content, water activity, and low microbial growth, leading to longer shelf‐life compared to the white bread. OFSP bread was also nutritionally beneficial, as it contained higher amounts of β‐carotene that persisted for 7 days. OFSP puree bread had a desirable textural property, especially as it was more cohesive and crumbled less. By visual observation, the white bread showed mold growth starting on the fourth day, while OFSP puree bread did not show spoilage until the sixth day. The OFSP puree bread is hence more shelf‐stable. However, the OFSP puree bread had less volume than the white bread and seemed smaller in size, despite near‐equal weight, although the bread's weight nearly equal. Storage at 7°C is recommended, as it preserved the quality (freshness and carotenoid content) of the samples. However, the storage in refrigeration increases the rate bread staling. This can be reverted by reheating the bread before consumption.

## CONFLICT OF INTEREST

The authors declare that they have no conflict of interest.

## ETHICAL STATEMENT

The study did not involve any human or animal testing.
